# Inhibition of platelet-derived growth factor signalling induces autophagy in malignant glioma cells

**DOI:** 10.1038/sj.bjc.6601605

**Published:** 2004-03-02

**Authors:** H Takeuchi, T Kanzawa, Y Kondo, S Kondo

**Affiliations:** 1 Department of Neurosurgery, The University of Texas MD Anderson Cancer Center, 1515 Holcombe Blvd., Unit 64, Houston, TX 77030, USA

**Keywords:** platelet-derived growth factor, neutralising antibody, glioma, autophagy

## Abstract

Malignant gliomas highly coexpress platelet-derived growth factor (PDGF) and its receptor, suggesting the presence of an autocrine loop. Therefore, disruption of PDGF ligand/receptor complex represents a promising strategy for the treatment of malignant gliomas. However, the mechanisms of the antitumour effect exerted by the inhibition of PDGF-mediated cell growth remain unclear. In the present study, using anti-PDGF neutralising antibody, we investigated the effect of the inhibition of PDGF signalling on malignant glioma U87-MG, D54, and T98G cells with high levels of PDGF-A and -B. As a control, normal fibroblast MRC5 cells expressing low levels of PDGF-A and -B were used. Treatment with anti-PDGF neutralising antibody did not affect the expressions of PDGF-A, PDGF-B, and Akt, but suppressed the level of phosphorylated Akt in tumour cells, indicating the inhibition of PDGF signalling. The cell viability of all malignant glioma cells tested in this study was significantly inhibited in a time-dependent manner following the treatment compared to that of fibroblast cells (*P*<0.02 to <0.05). The antitumour effect of anti-PDGF antibody was suppressed by the activation of Akt and enhanced by the downregulation of Akt. Interestingly, the inhibition of PDGF signalling induced the development of acidic vesicular organelles and the autophagosome membrane association of the microtubule-associated protein light chain 3, which are characteristic of autophagy, in malignant glioma cells, while apoptotic cell death was not observed. Together these findings imply a new concept of autophagy for PDGF autocrine inhibition in malignant gliomas.

Malignant gliomas are the most common brain tumour, but the cure rate and the life expectancy after the diagnosis of this tumour is very limited in spite of recent multidisciplinary advancement in radical surgery techniques assisted by microsurgery, imaging device, radiation therapy, and chemotherapy (Latif *et al*, 1998). Obviously, a novel treatment strategy of this tumour is required for prolonged survival.

Malignant tumour cells are well characterised by the unfettered reproduction through cell division of their progeny and themselves. One of the important factors that consist of this phenomenon is the autocrine signalling of growth factors (Vassbotn *et al*, 1993). The coexpression of platelet-derived growth factor (PDGF) and its receptor (PDGFR) is frequently detected in a variety of tumours including malignant glioma (Maxwell *et al*, 1990; Hermanson *et al*, 1992; Guha *et al*, 1995), lung cancer (Antoniades *et al*, 1992), and sarcoma (Smits *et al*, 1992), suggesting the existence of PDGF autocrine loop. Platelet-derived growth factor, a potent mitogen for glial cells, vascular smooth muscle cells, and fibroblasts, exists as disulphide-linked dimers of four homologous polypeptide chains, PDGF-A and -B, and the recently identified PDGF-C and PDGF-D (Ross *et al*, 1986; Deuel, 1987; Li *et al*, 2000; Bergsten *et al*, 2001; Gilbertson *et al*, 2001; LaRochelle *et al*, 2001). Platelet-derived growth factor exerts its biological activity by binding to structurally similar *α*- or *β*-PDGFRs. Ligand-induced receptor dimerisation leads to increased tyrosine kinase activity, triggering PDGF signal transduction molecules such as phosphatidylinositol 3-kinase (PI3K)/Akt kinase (Yu *et al*, 2003). The importance of PDGF signalling in malignant gliomas is demonstrated by recent investigation that PDGF autocrine stimulation in the brain of neonatal mice results in the formation of gliomas (Uhrbom *et al*, 1998). Therefore, the inhibition of PDGF/PDGFR complex is expected to represent a therapeutic efficacy for malignant gliomas. To block PDGF signalling, neutralising antibody to PDGF (Pollack *et al*, 1991; Fleming *et al*, 1992; Vassbotn *et al*, 1994), dominant-negative mutations of either PDGF or PDGFR (Vassbotn *et al*, 1993; Shamah *et al*, 1993; Strawn *et al*, 1994), or the tyrosine kinase inhibitors such as STI-571 (Kilic *et al*, 2000) or CT52923 (Lokker *et al*, 2002) was used, and the antitumour effect *in vitro* and *in vivo* was demonstrated. However, the mechanisms regulating the antitumour effect of blocking PDGF signal transduction pathways on malignant glioma cells remain unclear.

In the present study, using neutralising antibody to PDGF, we investigated the effect of the inhibition of PDGF signalling on malignant glioma cells with high levels of PDGF and fibroblast cells with low levels of PDGF. We demonstrate that the inhibition of PDGF signalling induces autophagy, but not apoptosis in tumour cells, while fibroblast cells are resistant to treatment with anti-PDGF neutralising antibody. Autophagy is a term used to describe a process of protein degradation in response to nutrient deprivation (Klionsky and Ohsumi, 1999; Kim and Klionsky, 2000). Recent studies show that autophagy is induced in tumour cells following radiation or chemotherapeutic agents (Bursch *et al*, 1996; Paglin *et al*, 2001; Kanzawa *et al*, 2003). Our results suggest a novel function of disruption of PDGF signalling on tumour cells.

## MATERIALS AND METHODS

### Reagents

Anti-human PDGF neutralising antibody (goat) was purchased from R&D systems (Minneapolis, MN, USA). A control antibody, IgG from goat serum was purchased from SIGMA (St Louise, MO, USA). Acridine orange was obtained from Polysciences (Warrington, PA, USA). Activated recombinant Akt was purchased from Upstate Biotechnology (Lake Placid, NY, USA).

### Cell culture

Human malignant glioma U87-MG and T98G cells and human normal fibroblasts MRC5 were purchased from American Tissue Culture Collection (Rockville, MD, USA). Human malignant glioma D54 cells were gifted by Dr F Lang (University of Texas MD Anderson Cancer Center, Houston, TX, USA). Cells were cultured in Dulbecco's modified Eagle's medium (DMEM, Invitrogen, Carlsbad, CA, USA) supplemented with 10% heat-inactivated fetal bovine serum, penicillin (50 U ml^−1^), and streptomycin (50 mg ml^−1^).

### Cell viability assay

The effect of anti-PDGF neutralising antibody on cells was determined by using a trypan blue dye exclusion assay as described previously (Komata *et al*, 2000). Cells were seeded at 5 × 10^3^ cells well^−1^ (0.1 ml) in 96-well flat-bottomed plates (Corning, Corning, NY, USA) and incubated overnight at 37°C. Then, the cells were daily treated with anti-PDGF antibody or goat IgG at a concentration of 1 *μ*g ml^−1^ for 3 days. For experiments using a recombinant active Akt or a recombinant adenovirus carrying dominant-negative Akt (AdDNAkt), cells were treated with anti-PDGF antibody or goat IgG in the presence of either a recombinant active Akt (50 ng) or AdDNAkt at a multiplicity of infection (MOI) of 20. The number of viable cells in each well was counted. Survival fractions were calculated from the mean cell viability of treated cells.

### Western blot

Cells were lysed in NP-40 lysis buffer (0.5%. NP-40, 140 mM NaCl, 10 mM Tris-HCl (pH 7)), supplemented with 3 mM MgCl_2_, 2 mM phenylmethylsulphonyl fluoride, and 5 mM dithiotheitol at 4°C for 30 min. The cells were harvested and centrifuged at 14 000 **g** for 30 min at 4°C to remove cellular debris. The supernatant was collected and protein content was measured using BCA Protein assay reagent (Pierce, Rockford, IL, USA). Equal amounts of the protein (40 *μ*g) from each sample were separated through 8–16% SDS–polyacrylamide gel electrophoresis and transferred to nitrocellulose membrane, and blocked with 5% nonfat dry milk in TBST (1 Tris-buffered saline+0.1% Tween 20) at room temperature for 1 h. The membrane was incubated overnight at 4°C with a primary antibody diluted in 5% nonfat dry milk in TBST. Primary antibody to actin (diluted at 1 : 500) was purchased from SIGMA, and antibodies to PDGF-A and -B (diluted at 1 : 200) were purchased from Santa Cruz (Santa Cruz, CA, USA). Primary antibody to Akt (diluted at 1 : 1000) was purchased from Cell Signalling Technology (Beverly, MA, USA), and antibody to phosphorylated Akt (Thr308) (diluted at 1 : 1000) was purchased from Upstate (Lake Placid, NY, USA).

The membrane was washed and then incubated for 1 h at room temperature with secondary antibody. Bound antibody was detected using enhanced chemiluminescence's reagent (ECL Western Blotting System; Amersham Bioscience Corp., Piscataway, NJ, USA).

### Apoptosis detection assay

To determine if apoptotic cells are observed in treated cells, we performed the terminal deoxynucleotidyl transferase-mediated dUTP nick end label (TUNEL) assay using ApopTag kit (Invitrogen) as described previously (Komata *et al*, 2000). Moreover, to determine whether treated tumour cells display apoptotic morphology, tumour cells were stained with Hoechst 33258 (8 *μ*g ml^−1^) as described previously (Kondo *et al*, 1995). Cisplatin (5 *μ*g ml^−1^) was used as a positive control to induce apoptosis (Kondo *et al*, 1995).

### Supravital cell staining with acridin orange

Cells were plated at the density of 2 × 10^5^ cells ml^−1^ for 1 day prior to treatment and incubated overnight at 37°C. Then, the cells were treated with anti-PDGF antibody or goat IgG, respectively. This anti-PDGF antibody or goat IgG treatment was performed every 24 h for 3 days. Then acridine orange was added at a final concentration of 1 *μ*g ml^−1^ for a period of 15 min. The cells were collected by trypsinisation, and green (510–530 nm) and red (>650 nm) fluorescence emission from cells illuminated with blue (488 nm) excitation light was measured with FACScan using CellQuest software as described previously (Paglin *et al*, 2001).

### Autophagy-specific staining

Recently, the green fluorescent protein (GFP)-tagged microtubule-associated protein light chain 3 (LC3)-expressing cells were used to demonstrate the induction of autophagy (Kabeya *et al*, 2000; Mizushima *et al*, 2003). Green fluorescent protein-LC3 cells presented a diffuse distribution under control, while a punctate pattern of GFP-LC3 was increased in number and fluorescence intensity by autophagy. Therefore, using the GFP-LC3 expression vector kindly supplied by Dr N Mizushima and Dr T Yoshimori, (National Institute for Basic Biology, Japan), U87-MG cells expressing GFP-LC3 (GFP-LC3-U87-MG cells) were established. Briefly, transfection was performed on 100-mm plates with 10 *μ*g plasmid DNA/plate with GenePorter (Gene Therapy System Inc., San Diego, CA, USA) according to the manufacturer's instructions. After overnight exposure, cells were washed three times with phosphate-buffered saline and cultured in complete medium. At 48 h after the medium change, cells were selected in medium containing 400 *μ*g ml^−1^ of G418 (GIBCO BRL) and G-418-resistant clones were established. To detect the localiszation of LC3, GFP-LC3-U87-MG cells were cultured on the chamber slide dish (Fisher Inc.). After exposure to anti-PDGF neutralising antibody or goat IgG for 3 days, cells were fixed in 4% paraformaldehyde and analysed by fluorescence microscopy.

### Statistical analysis

The data were expressed as means±s.d. Statistical analysis was performed by using Student's *t*-test (two-tailed). The criterion for statistical significance was taken as *P*<0.05.

## RESULTS

### Expression of PDGF in malignant glioma cells and normal fibroblast cells

**Figure 1:**
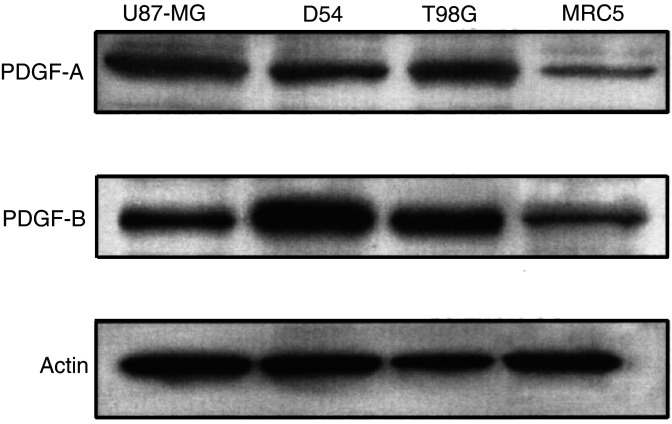
Expression of PDGF in malignant glioma cells and normal fibroblast cells. Aliquots of 40 *μ*g of protein extract from U87-MG, D54, T98G, or MRC5 cells were used for immunoblotting assay using anti-PDGF-A or -PDGF-B antibody. The anti-*β*-actin monoclonal antibody was used for protein-loading equivalence. Data shown are representative of three independent experiments.

To determine the expression levels of PDGF in malignant glioma cells and fibroblast cells, the Western blot was performed. As shown in Figure 1

, PDGF-A and -B were highly positive in U87-MG, D54, and T98G cells, while the protein levels of PDGF-A and -B were low in MRC5 cells. The intensity of PDGF-A or -B expression in U87-MG, D54, and T98G cells was 22.1- to 32.6-fold or 15.2- to 57.4-fold higher than in MRC5 cells, respectively. These results indicate that U87-MG, D54, and T98G cells expressed high levels of PDGF-A and -B, while the expression levels were very low in MRC5 cells.

### Anti-PDGF antibody reduces the level of phosphorylated Akt in malignant glioma cells

**Figure 2:**
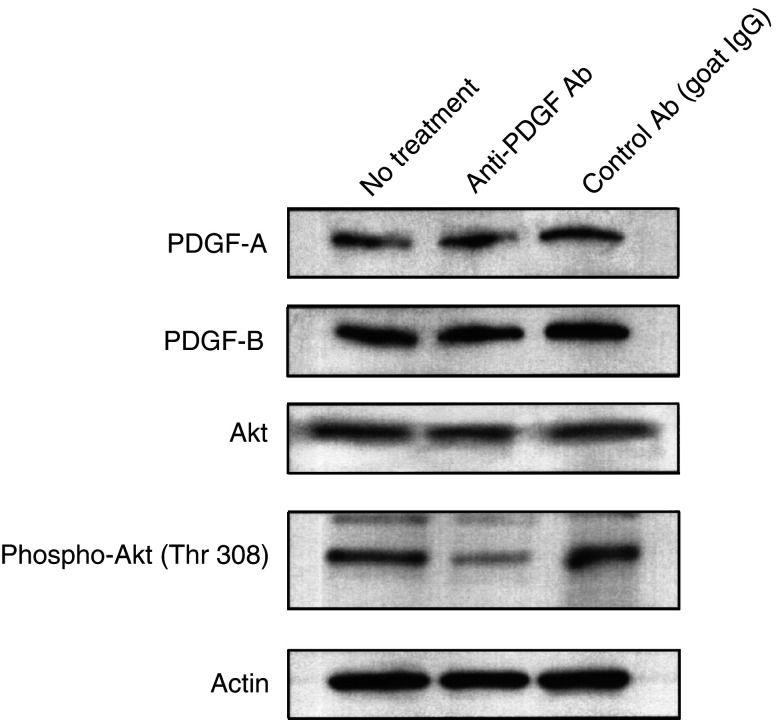
Effect of neutralising anti-PDGF antibody on expression of PDGF-A, PDGF-B, Akt, or phosphorylated Akt in malignant glioma cells. Aliquots of 40 *μ*g of protein extract from U87-MG cells treated with anti-PDGF or control antibody (1 *μ*g ml^−1^) for 24 h were used for immunoblotting assay using anti-PDGF-A, -PDGF-B, -Akt, or -phosphorylated Akt (Thr 308) antibody. The anti-*β*-actin monoclonal antibody was used for protein-loading equivalence. Data shown are representative of three independent experiments.

To investigate the ability of anti-PDGF antibody to neutralise PDGF and inhibit PDGF signalling, U87-MG cells were cultured in the presence of anti-PDGF antibody or control antibody (goat IgG) (1 *μ*g ml^−1^) for 24 h. As shown in Figure 2

, the Western blot revealed that treatment with anti-PDGF antibody or control antibody did not affect the expression of PDGF-A or -B in U87-MG cells. Growth signal originated from PDGF/PDGFR is transduced via PI3K/Akt pathway (Adi *et al*, 2001). Therefore, to determine if treatment with anti-PDGF antibody inhibits PDGF signalling in malignant glioma cells, the phosporylation of Akt was examined by Western blot. As shown in Figure 2, U87-MG cells displayed constitutive Akt phosphorylation (Thr 308) that was inhibited to 36.9% of the no treatment by treatment with anti-PDGF antibody, although the level of Akt protein was not influenced. On the other hand, the effect of control antibody on phosphorylated Akt was minimal. Similar results were observed in other glioma cells (data not shown). These results indicate that treatment with anti-PDGF antibody did not affect the expression levels of PDGF-A, PDGF-B, and Akt, but reduced phosphorylated Akt, suggesting that PDGF signalling was inhibited in malignant glioma cells.

### Anti-PDGF antibody reduces cell viability in malignant glioma cells, but not in normal fibroblast cells

**Figure 3:**
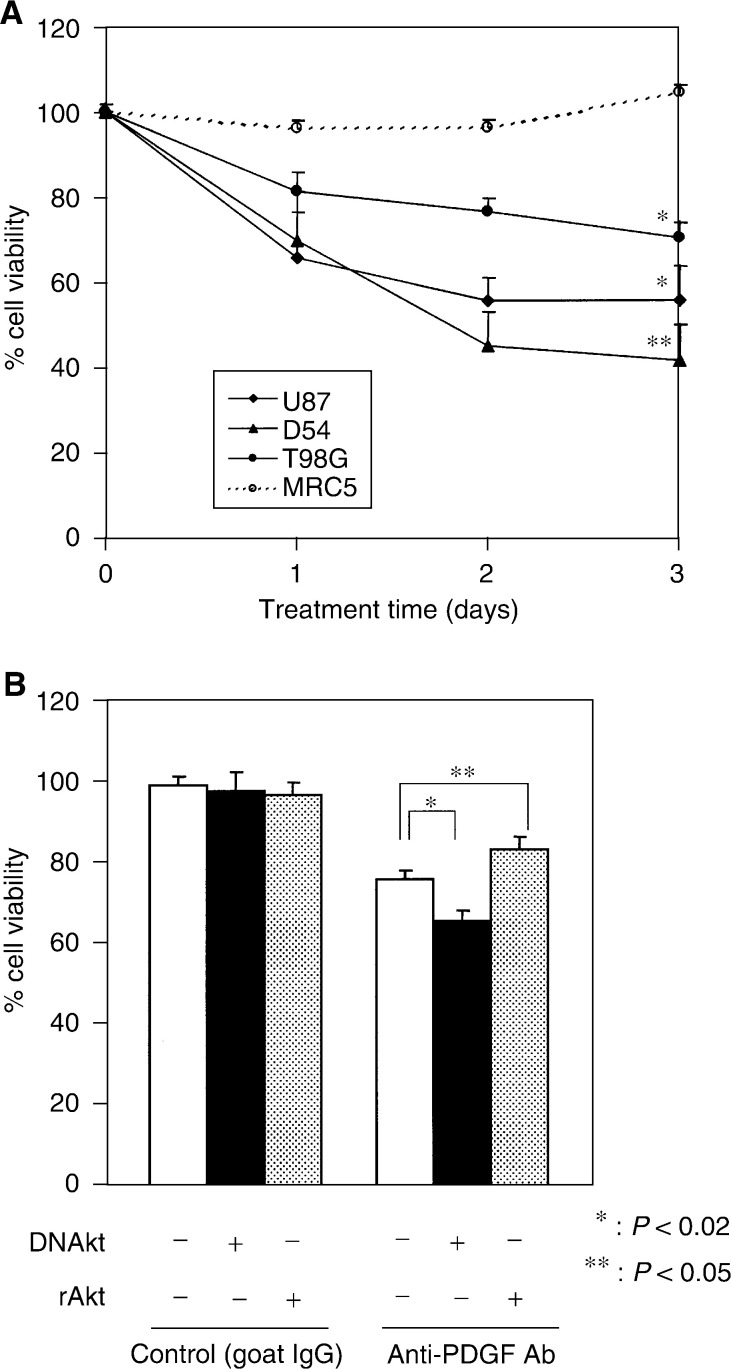
Effect of neutralising anti-PDGF antibody on cell viability of malignant glioma cells. (**A**) Inhibition of cell viability in malignant glioma cells following the treatment with anti-PDGF antibody. Tumour cells (U87-MG, D54, and T98G) and fibroblast MRC5 cells were seeded at 5 × 10^3^ cells well^−1^ (0.1 ml) in 96-well flat-bottomed plates and incubated overnight at 37°C. After exposure to anti-PDGF or control antibody (1 *μ*g ml^−1^) for 1–3 days, the cells were trypsinised and the number of viable cells was counted. The viability of the cells treated with control antibody was regarded as 100%. Results shown are the means±s.d. of three independent experiments. ^*^P<0.05, ^**^P<0.02. (**B**) Effect of extrinsic activation or inactivation of Akt on cell viability. U87-MG cells were treated with anti-PDGF antibody or control antibody in the presence of recombinant active Akt (50 ng) or adenovirus-mediated dominant-negative Akt (20 MOI) for 3 days. Results shown are the means±s.d. of three independent experiments. ^*^P<0.02, ^**^P<0.05.

As PDGF-PDGFR autocrine signalling plays an important role in the tumour cell survival, treatment with anti-PDGF antibody might reduce growth of malignant glioma cells by blocking PDGF-PDGFR autocrine loop. Therefore, we examined the effect of anti-PDGF antibody on cell viability of malignant glioma cells with high levels of PDGF. As shown in Figure 3A

, cell proliferation of U87-MG, D54, and T98G cells treated with anti-PDGF antibody was significantly reduced to 41 to 74% of the cells treated with control antibody compared to that of MRC5 cells (*P*<0.02 to <0.05). To ensure that reduced viability of malignant glioma cells is due to the inhibition of PDGF signalling, cells were further treated with anti-PDGF antibody in the presence of a recombinant active Akt or a recombinant adenovirus-expressing dominant-negative Akt (AdDNAkt). As shown in Figure 3B, the activation of the Akt kinase pathway by the addition of a recombinant active Akt conferred the resistance of U87-MG cells to treatment with anti-PDGF antibody (*P*<0.05). In contrast, the downregulation of Akt activity following infection of AdDNAkt sensitised tumour cells to the treatment (*P*<0.02). The presence of a recombinant active Akt or AdDNAkt did not affect the cell viability of tumour cells treated with control IgG. These results suggest that treatment with anti-PDGF antibody reduced the cell growth of tumour cells via the downregulation of Akt activity, while normal cells were insensitive to the treatment.

### Anti-PDGF antibody does not induce apoptosis

**Figure 4:**
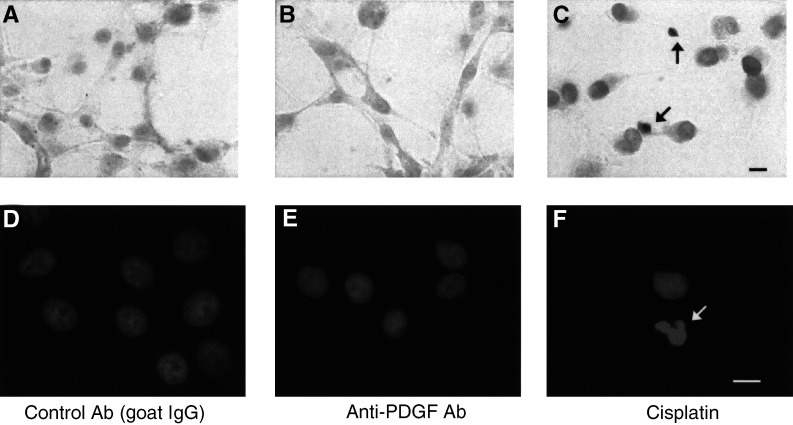
Apoptosis detection assays for malignant glioma cells treated with anti-PDGF antibody. After the treatment with anti-PDGF (**B**) or control antibody (**A**) (1 *μ*g ml^−1^) for 72 h, U87-MG cells were fixed, labelled with Br-dUTP, and stained with anti-Br-dUTP antibody for TUNEL analysis. As a positive control for apoptosis, U87-MG cells were treated with cisplatin (5 *μ*g ml^−1^) for 2 days (**C**). To evaluate apoptotic morphological changes in U87-MG cells treated with control (**D**), anti-PDGF antibody (**E**), or cisplatin (**F**), the cells were fixed and stained with Hoechst 33258 (8 *μ*g ml^−1^). Arrows indicate representative apoptotic cells. Data shown are representative of three independent experiments. Bar, 10 *μ*m.

To determine whether the effect of anti-PDGF antibody against tumour cell proliferation is due to the induction of apoptosis, the TUNEL assay and Hoechst 33258 staining were performed for U87-MG cells treated with anti-PDGF or control antibody. As shown in Figure 4

, a significant number of TUNEL-positive (c) or DNA-condensed or -fragmented cells (f) were detected following the treatment with cisplatin (5 *μ*g ml^−1^) for 2 days. However, there were almost no TUNEL-positive or morphologically apoptotic cells in U87-MG cells treated with anti-PDGF (Figure 4B or E) or control antibody (Figure 4A or D). These results indicate that the antitumour effect of the inhibition of PDGF signalling was not due to the induction of apoptosis.

### Anti-PDGF antibody develops acidic vesicular organelles (AVOs)

**Figure 5:**
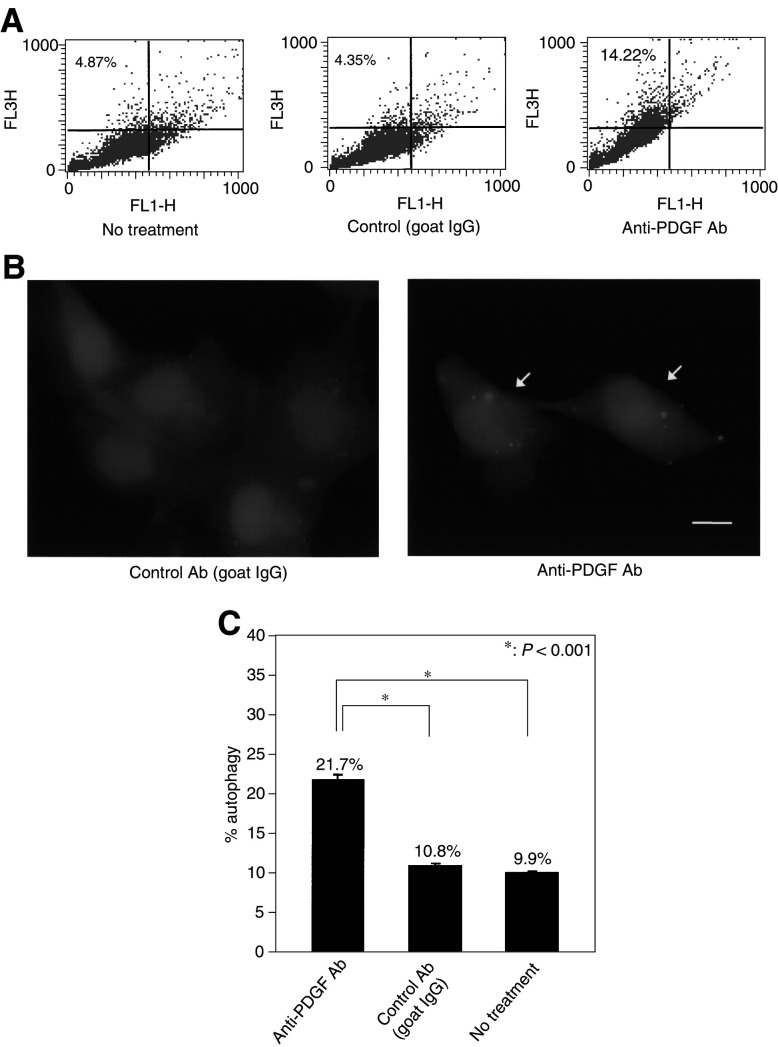
Induction of autophagy in malignant glioma cells following the treatment with anti-PDGF antibody. (**A**) Development of AVOs in U87-MG cells treated with anti-PDGF antibody. Detection of green and red fluorescence in acridine orange-stained cells was performed using FACS analysis. Tumour cells were treated with anti-PDGF or control antibody (1 *μ*g ml^−1^) for 3 days. FL1-H indicates green color intensity, while FL3-H shows red color intensity. Data shown are representative of three independent experiments. (**B**) Involvement of LC3 autophagosome membrane association in the inhibition of PDGF signalling. Green fluorescent protein-LC3-U87-MG cells were treated with anti-PDGF or control antibody (1 *μ*g ml^−1^) for 3 days and examined by fluorescence microscopy. Arrows indicate representative autophagic cells. Data shown are representative of three independent experiments. Bar, 10 *μ*m. (**C**) The percentage of cells showing punctate pattern of LC3 after treatment with anti-PDGF antibody. After the treatment with anti-PDGF or control antibody (1 *μ*g ml^−1^) for 3 days, the percentage of autophagy was quantified by counting the number of the cells showing the punctate pattern of LC3-GFP in 50 GFP-positive cells. Results shown are the means±s.d. of three independent experiments. ^*^P<0.001.

Recently, we and other investigators have demonstrated that radiation or some chemotherapeutic agents induce autophagy, but not apoptosis with increased autophagosomes in tumour cells (Bursch *et al*, 1996; Paglin *et al*, 2001; Kanzawa *et al*, 2003; Yao *et al*, 2003). Autophagy is characterised by the development of AVOs. To identify the development of AVOs in malignant glioma cells treated with anti-PDGF antibody, we used the lysosomotropic fluorescent dye acridine orange, a weak base that moves freely across biological membranes when uncharged. In acridine orange-stained cells, the cytoplasm and nucleolus fluoresce bright green and dim red, whereas acidic compartments fluoresce bright red (Paglin *et al*, 2001). As shown in Figure 5A

, anti-PDGF antibody increased the strength of the bright red fluorescence (*y*-axis) in U87-MG cells from 4.87 to 14.22%, indicating the development of AVO. On the other hand, significant development of AVO was not detected in U87-MG cells treated with control antibody (4.35%). These results indicate that the inhibition of PDGF signalling induces autophagic change in malignant glioma cells.

### Anti-PDGF antibody induces autophagosome membrane association of LC3

Although the induction of autophagy is usually associated with the increase in the number of acidic compartments (autolysosomes), the induction of AVO does mean the activation of the lysosomal system. Therefore, as a specific assay for autophagy, GFP-LC3-expressing vectors were used in several cases to demonstrate the induction of autophagy (Kabeya *et al*, 2000; Munafo and Colombo, 2001; Mizushima *et al*, 2003). Therefore, GFP-LC3-expressing U87-MG cells were treated with anti-PDGF or control antibody in order to observe the formation of autophagosome following the treatment. As shown in Figure 5B, GFP-LC3 presented diffuse distribution in tumour cells treated with control antibody. In contrast, after treatment with anti-PDGF antibody for 3 days, a punctate pattern of GFP-LC3 was detected in some cells. The percentage of cells showing this autophagic pattern of GFP-LC3 expression following the treatment with anti-PDGF antibody increased from 9.9% (no treatment) or 10.8% (control Ab) to 21.7% (*P*<0.001; Figure 5C). These results indicate that the inhibition of PDGF signalling formed autophagosome and promoted autophagy in U87-MG cells.

## DISCUSSION

In the present study, we demonstrate that the expressions of PDGF-A and -B were significantly higher in malignant glioma cells than non-tumoral fibroblast cells. It is consistent with the previous findings that malignant gliomas (anaplastic astrocytomas and the most malignant type, glioblastoma multiforme) overexpress the ligands PDGF-A and -B compared to low-grade gliomas or normal tissues (Maxwell *et al*, 1990; Hermanson *et al*, 1992; Guha *et al*, 1995). Moreover, the retrovirus-mediated expression of PDGF-B in the brain of neonatal mice results in the formation of astrocytomas (Uhrbom *et al*, 1998). These observations support the importance of PDGF signalling in malignant gliomas. Accordingly, it is reasonable to treat malignant gliomas with the inhibition of PDGF signalling. To date, several approaches for disruption of PDGF/PDGFR pathway are introduced. Using a neutralising anti-PDGF antibody or dominant-negative mutation of PDGF, PDGF signalling is inhibited, resulting in diminished DNA synthesis, the inhibition of tumour colony growth, and reversion of the transformed morphology of the tumour cells (Pollack *et al*, 1991; Fleming *et al*, 1992; Shamah *et al*, 1993; Vassbotn *et al*, 1993, 1994). In addition, treatment with the truncated PDGFR or the PDGFR tyrosine kinase inhibitors such as STI-1571 or CT52923 inhibited PDGFR signaling, and the *in vitro* and *in vivo* growth of malignant gliomas (Strawn *et al*, 1994; Kilic *et al*, 2000; Lokker *et al*, 2002). However, very little is known about what plays a central role in the antitumour effect observed following the inhibition of PDGF/PDGFR signalling pathway. We here demonstrate that the inhibition of PDGF signalling by a neutralising anti-PDGF antibody suppressed the proliferation of malignant glioma cells with high levels of PDGF through the inhibition of Akt activity, resulting in the induction of autophagy, but not apoptosis. In contrast, normal fibroblast cells expressing low levels of PDGF were resistant to the treatment with anti-PDGF antibody. To our knowledge, the present study is the first report demonstrating that autophagy is induced in malignant glioma cells by disruption of PDGF signalling.

Autophagy is characterised by the accumulation of autophagic vacuoles, and is well described as the degradation of normal proteins in response to nutrient deprivation or other types of stresses (Klionsky and Ohsumi, 1999; Kim and Klionsky, 2000). In the process of autophagy, cytoplasmic materials are sequestered by a membrane vacuole called autophagosome. Autophagosome then fuses with lysosome, where the materials inside are degraded by lysosomal hydrolases. Using this process, autophagy recycles molecules for biosynthetic reactions. In the present study, the inhibition of PDGF signalling induced the development of AVOs and increased punctate patterns of GFP-LC3 characteristic of autophagy. LC3 is a mammalian homologue of Apg8p/Aut7p essential for autophagy in yeast (Kabeya *et al*, 2000) and is recruited to the autophagosome membrane in the Apg5-dependent manner (Mizushima *et al*, 2001). Therefore, autophagosome membrane association of LC3 is a specific marker for autophagy (Kabeya *et al*, 2000; Munafo and Colombo, 2001; Mizushima *et al*, 2003). Recent investigations suggest that autophagy plays a defensive role in cancer therapy (Bursch *et al*, 1996; Paglin *et al*, 2001; Ogier-Denis and Codogno, 2003). Therefore, if it is the case, the inhibition of autophagy may enhance the antitumour effect of the inhibition of PDGF signalling.

PDGF autocrine signalling characterised by the coexpression of PDGF and PDGFR contributes to the development of a variety of tumours including malignant gliomas as a major signal pathway (Maxwell *et al*, 1990; Antoniades *et al*, 1992; Hermanson *et al*, 1992; Smits *et al*, 1992; Vassbotn *et al*, 1993; Guha *et al*, 1995). Secreted PDGF binds to extracellular domain of cell surface PDGFR, resulting in receptor dimerisation. Ligand binding is required for the stability of the receptor dimers and their activation. Platelet-derived growth factor receptor is classified as the receptor protein tyrosine kinases that are so named since they are activated when specific tyrosine residues on the intracellular domain of the receptor are phosphorylated. After dimerisation, phosphorylated tyrosine residues interact with Src homology 2 domains of intracellular signalling molecules including PI3K/Akt (Heidaran *et al*, 1993; Kazlauskas *et al*, 1993; DeMali *et al*, 1997; Rosenkranz and Kazlauskas, 1999). In the present study, anti-PDGF antibody reduced phosphorylation of Akt, indicating that PDGF signalling leading to PI3K/Akt activation is suppressed. The activation of Akt using activated recombinant Akt suppressed the antitumour effect of anti-PDGF antibody, while the inhibition of Akt with AdDNAkt augmented anti-PDGF antibody-induced cytotoxicity. These results suggest that the inhibition of Akt signalling might play an important role in the induction of autophagy in malignant glioma cells. Activated signalling molecules further transduce signal transduction pathways by activating downstream signalling molecules such as mitogen-activated protein kinase family members (Yu *et al*, 1994; Choudhury *et al*, 1997; Maudsley *et al*, 2000; Lokker *et al*, 2002). These signals enter the nucleus and stimulate the expression of a set of immediate-early response genes mediating PDGF-induced cellular processes such as cell cycle, migration, and transformation. On the other hand, there is accumulating evidence that autophagy is a multistep process and a variety of signalling kinases have been implicated in the upregulation or downregulation of autophagy (Bursch *et al*, 2000; Klionsky and Emr, 2000; Ogier-Denis and Codogno, 2003). In particular, PI3K is an important factor in autophagic process (Klionsky and Ohsumi, 1999; Kabeya *et al*, 2000; Kim and Klionsky, 2000; Mizushima *et al*, 2001; Munafo and Colombo, 2001; Paglin *et al*, 2001). Three classes of PI3K have been defined so far. Class I PI3K is involved in the inhibitory effect on autophagy, while the class III PI3K is involved in the sequestration of cytoplasmic material in autophagy (Petiot *et al*, 2000). Therefore, autophagy induced by the inhibition of PDGF signalling may be due to the inhibitory effect on class I PI3K and/or the stimulatory effect on class III PI3K. Further study is necessary to investigate the molecular mechanisms regulating the induction of autophagy in malignant glioma cells treated with anti-PDGF antibody.

In conclusion, the inhibition of PDGF signalling by a neutralising anti-PDGF antibody suppressed the proliferation of malignant glioma cells through the induction of autophagy, but not apoptosis. In contrast, normal fibroblast cells with low levels of PDGF were significantly resistant to the anti-PDGF antibody therapy. These findings provide strong evidence that the inhibition of PDGF signalling exhibits antitumour effect only on tumour cells and autophagy plays a key role in its efficacy.
